# Emphysematous Pyelonephritis Presenting as Pneumaturia and the Use of Point-of-Care Ultrasound in the Emergency Department

**DOI:** 10.1155/2019/6903193

**Published:** 2019-09-02

**Authors:** Natasha Brown, Paul Petersen, David Kinas, Mark Newberry

**Affiliations:** ^1^Mount Sinai Medical Center, 4300 Alton Rd., Miami Beach, FL 33140, USA; ^2^FIU Herbert Wertheim College of Medicine, 11200 SW 8th St., Miami, FL 33199, USA

## Abstract

Emphysematous pyelonephritis (EPN) is a rare form of pyelonephritis causing a severe infection of the renal system that includes gas in the renal parenchyma, collecting system and surrounding tissue often presenting with sepsis. We report the case of a 60-year-old male with poorly controlled insulin dependent diabetes mellitus who presented with abdominal pain, nausea, vomiting, and “peeing air.” CT scan revealed air extending from the left renal parenchyma, perinephric fat and into the bladder, consistent with emphysematous pyelonephritis. Bedside point-of-care ultrasound (POCUS) subsequently revealed dirty shadowing and reverberation artifacts in the left kidney and the bladder consistent with gas in the urinary collecting system. By understanding the identifying artifacts seen with EPN, reflective shadow and reverberation artifact, the emergency physician may be alerted to the diagnosis sooner. Often this illness presents similarly to simple, acute pyelonephritis or undifferentiated sepsis. Therefore, POCUS allows for real time consideration of this condition while in the emergency department and thus prompter time to treatment.

## 1. Introduction

Emphysematous pyelonephritis (EPN) is a rare form of pyelonephritis causing a severe infection of the renal system that includes gas in the renal parenchyma, collecting system and surrounding tissue often presenting with fever, dysuria, and sepsis of renal origin. The clinical course of EPN is life threatening if not promptly recognized and treated. We present a case of emphysematous pyelonephritis that was diagnosed by CT scan but shows specific point-of-care ultrasound findings in a 60-year-old diabetic male presenting with vague complaints of fatigue and vomiting. This case emphasizes how point-of-care ultrasound (POCUS) can help in the differential diagnosis and potentially a more rapid recognition of a serious life-threatening disease.

## 2. Case Presentation

A 60-year-old male with poorly controlled insulin dependent diabetes mellitus, noncompliant with his medication presented to our emergency department complaining of generalized abdominal pain, worse in the right upper quadrant (RUQ), nausea, nonbloody, nonbilious vomiting, and fatigue for two days. The patient's initial vital signs showed a heart rate of 103 beats/min, temperature of 36.8°C orally, respiratory rate of 24 breaths/min, a blood pressure of 145/88 mmHg and an oxygen saturation of 100% on room air. The patient's point-of-care glucose was 514 mg/dl. The patient denied prior medical treatment, however a family member endorsed that he had not been taking his insulin injections or sulfonylurea medications as prescribed. Upon further questioning the patient admitted to “peeing air”, described as “farting out of my penis” for 3 weeks and no urine production for the last day.

The physical examination revealed a weak, tachypneic, toxic appearing male slow to answer questions who appeared his stated age. He was lethargic, oriented to person, place, and time, with dry oral mucosa, poor skin turgor, fruity smelling breath, and mild tenderness to palpation of bilateral upper abdominal quadrants without costovertebral angle tenderness. The rest of the exam was age appropriate and within normal limits. Initially, our differential diagnosis included sepsis, diabetic ketoacidosis, hyperosmolar hyperglycemic state, and nephrolithiasis/ureterolithiasis. The patient had a prior cholecystectomy, making gallbladder pathology less likely.

Laboratory results revealed a leukocytosis of 14.44 × 10^3^/*µ*L (4.80 × 10^3^/*µ*L–10.80 × 10^3^/*µ*L) with a significant left shift, bandemia of 26% (0%–10%), thrombocytopenia of 38000 × 10^3^/*µ*L (150 × 10^3^/*µ*L–450 × 10^3^/*µ*L), and a lactate of 4.0 mmol/L (0.4–2.0 mmol/L). The patient was found in new onset renal failure as indicated by a glomerular filtration rate of 14 mL/min/1.73 m^2^ (>60 mL/min/1.73 m^2^; GFR = 186 × Serum Cr^−1.154^ × age^−0.203^ × 1.212 (if patient is black) × 0.742 (if female)) [1] and a creatinine of 4.4 mg/dl (0.70 mg/dl–1.30 mg/dl) which was previously recorded at 0.8 mg/dl. Urinalysis revealed significant pyuria with 100 mg/dl proteinuria. The patient was immediately recognized to be septic and given broad spectrum antibiotics with aggressive fluid resuscitation. The patient was given a 30 cc/kg bolus of normal saline and intravenous doses of 3.375 g piperacillin-tazobactam and 600 mg clindamycin pending results of urine and blood cultures. We were concerned that this patient with abdominal pain and sepsis in an uncontrolled diabetic had a life-threatening abdominal source of infection.

**Figure 1:**
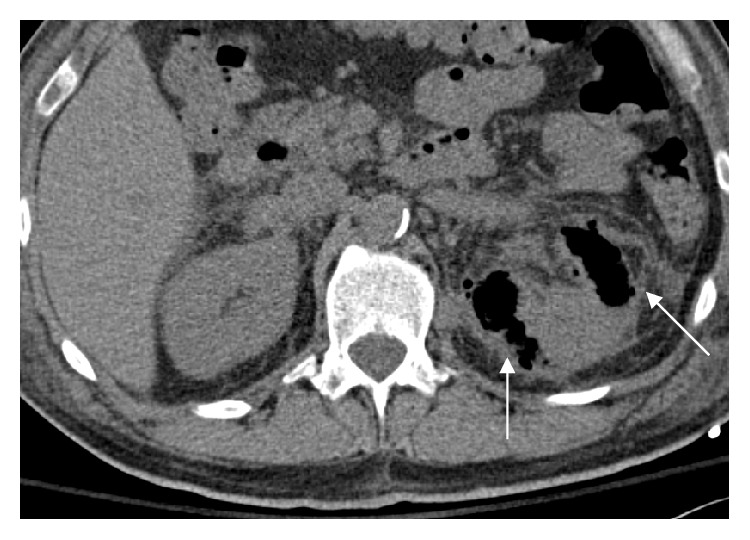
Axial noncontrast CT scan of the abdomen showing air (arrows) in the left renal parenchyma.

**Figure 2:**
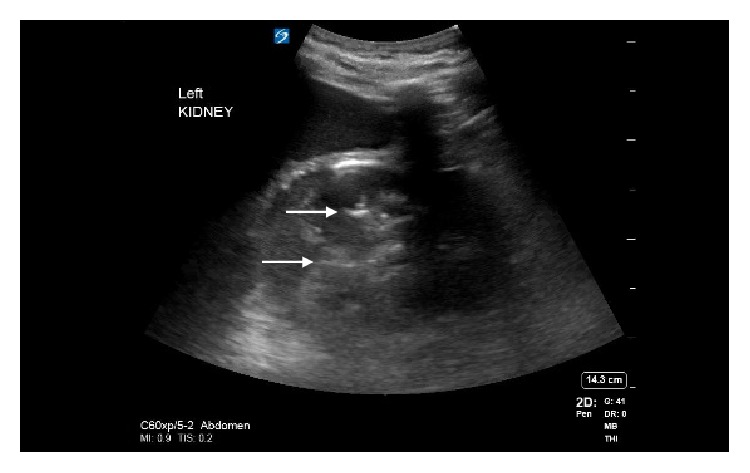
Longitudinal ultrasonography of the left kidney showing a- lines (arrows), indicating free air in the renal parenchyma.

**Figure 3:**
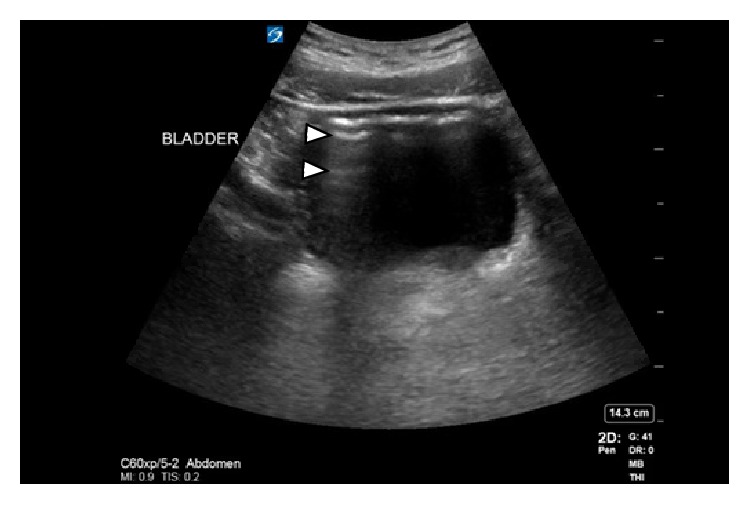
Transverse ultrasound of the bladder showing reverberation artifacts (triangles) in the dirty shadow cast by the tissue–gas interface in the urinary bladder.

A 64-slice detector computerized tomography (CT) scan without intravenous contrast with approximately CTVI 10 mGy dose of radiation was performed which revealed extensive left perinephric fat stranding with gas extending from the left renal parenchyma, perinephric fat, left ureter, and into the bladder (Figure 1) revealing the diagnosis of emphysematous pyelonephritis. There was no evidence of obstruction secondary to renal calculi, or urinary retention. Then, for educational purposes, a bedside ultrasound of the left kidney and bladder was performed using a Sonosite X-porte machine with a curvilinear C60x 5-2 MHz probe on the system presetting for abdominal imaging. The left kidney showed reverberation artifacts (white arrows), caused by air in the renal pelvis (Figure 2). Subsequent scanning of the urinary bladder showed a fluid–gas interface in the left upper quadrant of the bladder image (Figure 3), also demonstrated reverberation artifact (triangular pointers). This is consistent with abnormal gas in the urinary collecting system. Urgent urologic consultation was obtained, recommending continued conservative management with fluid resuscitation and antibiotics. The patient was admitted to the intensive care unit. Urology assessed the patient to be a high-risk candidate for an emergent operative nephrectomy. In consultation with interventional radiology, the patient was managed nonoperatively with a percutaneous nephrostomy tube and left ureteral catheterization performed by an interventional radiologist. The urine culture grew pan-sensitive *E. coli* and blood cultures returned negative. Subsequently, the antibiotic regimen was changed to IV ceftriaxone. He was later discharged on oral ciprofloxacin for 4 weeks with the nephrostomy tube in place and returned two months later for an outpatient left nephrectomy secondary to a fibrotic kidney with multiple adhesions.

## 3. Discussion

EPN is a rare and complicated bacterial infection of the kidney that causes severe necrosis in the renal parenchyma, urinary collecting system, and surrounding perinephric tissue. The clinical course of EPN is often critical and has a high mortality if not diagnosed and treated early mainly due to complications from septic shock [2]. Unfortunately, this disease is poorly reported in the literature as it is usually in the form of case reports. The most commonly associated factor is diabetes mellitus; as many as 95% of patients with EPN may have underlying uncontrolled diabetes [2, 3]. The leading explanation for the increased susceptibility to this disease in diabetic patients is that decreased blood flow and high glucose concentrations facilitate anaerobic bacterial growth. This disease has also been shown to be associated with drug abuse, alcoholism, and neurogenic bladder [3]. According to a review of multiple small studies, the female predominance of EPN may be as pronounced as 6 : 1, thought to be due to the increased susceptibility to urinary tract infections [2]. The major causative organism for EPN is the same as for urinary tract infection's in general, which is *E. coli* [2]. Gas is formed through the fermentation of glucose and lactate which results in the production of high levels of carbon dioxide and hydrogen gasses at the site of the infection [2].

The presentation of EPN can be insidious and similar to noncomplicated pyelonephritis with the most common symptoms being abdominal pain (94.11%), fever (83.2%) and dysuria (74.5%) [3]. Abdominal pain is generally thought to include flank pain and/or tenderness and its absence should raise a list of alternative diagnoses [3, 4]. Atypical presentations are much more common in uncontrolled diabetics such was seen in our patient who presented with right upper quadrant pain.

Shock, defined as a systolic blood pressure of less than 90 mmHg, was only present in 16% of patients in one retrospective study of 74 patients [4]. Positive urinalysis for pyuria was reported to be the most common lab abnormality, which was 79%–100% sensitive [4–7]. Shock, altered mental status and elevated creatinine are described to be associated with a higher mortality [5, 6].

The more widespread use of CT scan and ultrasound has seen an increasing incidence of EPN [6]. There is typically little role for CT or ultrasound in a simple pyelonephritis as it is a clinical diagnosis, however if the patient has comorbidities, is toxic appearing, or is not responsive to treatment, imaging may reveal complications such as obstructive pathology, perinephric fluid collections and/or gas. In the case of EPN, diagnosis is typically made by CT scan. There are 4 distinct radiographic classifications; Class 1: gas in the collecting system only (emphysematous pyelitis); Class 2: gas in the renal parenchyma without extra-renal extension; Class 3A: gas extension, or abscess formation in the perinephric area; Class 3B: involvement of para-renal space; Class 4: bilateral EPN or a solitary kidney with EPN [7]. Our patient therefore had progressed to Class 3A. Management previously included emergent nephrectomy, however new advances in percutaneous technology in conjunction with medical management have decreased mortality to as low as 13.5% [8]. Medical management typically includes aggressive intravenous fluid resuscitation and antibiotic therapy.

The increased utilization of POCUS in the emergency department has allowed for real time clues to the source of infection. Specific POCUS applications are utilized based on a patient's presentation to answer specific clinical questions and help guide management and patient disposition. More guided diagnostic algorithms have been described as well, for example, to evaluate medical hypotension (Rapid Ultrasound in Shock (RUSH) exam). Recently a POCUS guided protocol for sepsis was proposed, using first echocardiography, and inferior vena cava compressibility to assess cardiac contractility and fluid status, then focusing on source [9]. Renal ultrasound gives emergency physicians the ability to demonstrate hydronephrosis, renal enlargement, edema, and collections of fluid or air [10].

**Figure 4:**
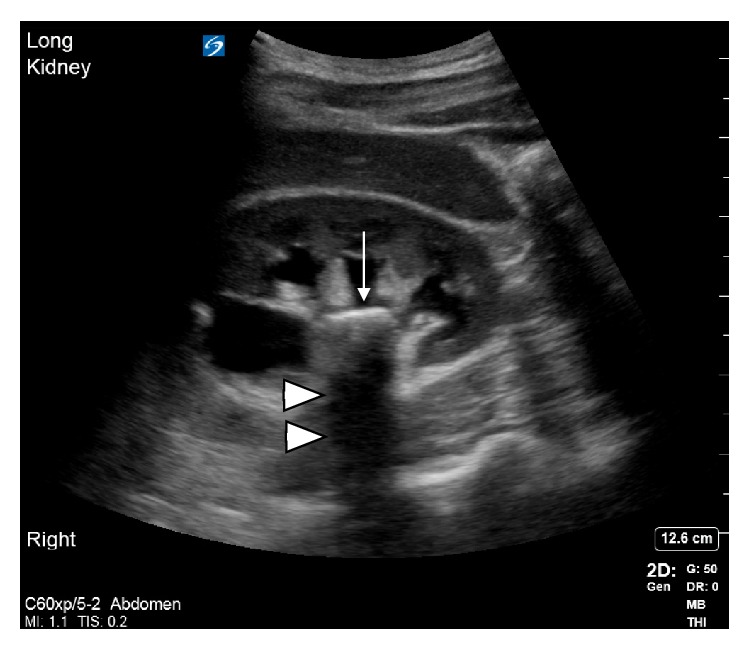
Longitudinal view of a right kidney showing a calculus (white arrow) with posterior clean shadowing (triangles).

In cases of EPN, ultrasound may illustrate intrarenal, intraurethral, or intracystic air artifacts. These artifacts can include “dirty shadowing” and reverberation artifacts commonly referred to as “a-lines.” To understand dirty shadowing, one must first understand clean shadowing. Clean shadowing is created by sound wave absorbing structures such as bone [11]. Because of this absorption, a shadow is cast deep to the structure (Figure 4). The ultrasound waves do not penetrate the deeper structures.

**Figure 5:**
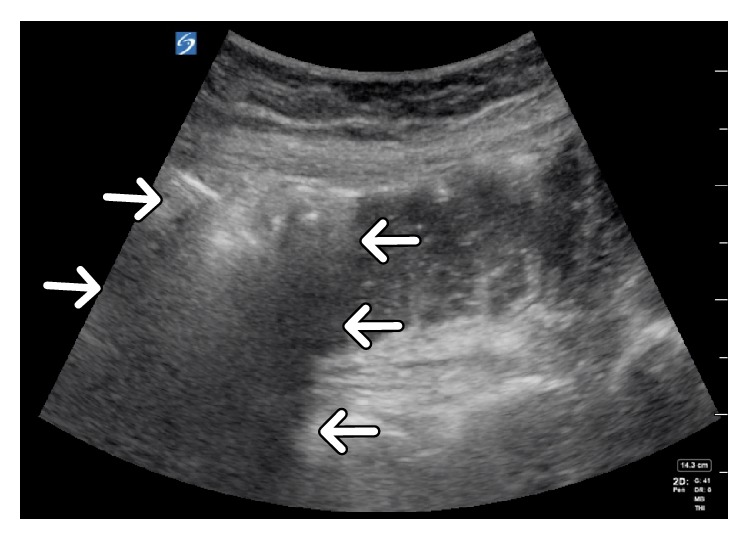
Dilated loop of bowel with adjacent area of “dirty shadowing” (between the arrows).

In contrast, dirty shadowing has a distinct appearance with irregular borders because of a tissue–gas interface formed within the body. Since there is a large acoustic impedance difference between the tissue and gas it mostly reflects rather than absorbs ultrasound waves, therefore, a tissue–gas interface creates an irregular hyper-echoic image with reverberation artifacts that form deep to the interface [12]. A nonpathologic example of dirty shadowing is a normal gas filled bowel (Figure 5). The tissue–gas interface creates one highly reflective surface and in the presence of a second highly reflective parallel surface, the echoes generated from a primary ultrasound beam may be repeatedly reflected back and forth before returning to the transducer for detection. These repetitive, regularly spaced artifactual horizontal stripes that appear deep in an air-filled structure are a form of reverberation artifacts called a-lines [12]. A good nonpathologic example of a-lines is in normal lung [11]. In EPN, a-lines seen in the collecting system or renal parenchyma tissue are by definition pathologic because they are an illustration of gas filled structure tissue interface within the renal collecting system (Figures 2 and 3).

To summarize, both clean and dirty shadowing artifacts are due to attenuation of sound. The difference is, clean shadowing is due to the interface of a substance such as tissue and a strongly absorbing material such as bone or calcified stone while dirty shadowing is due to a high degree of reflection at a gas–tissue interface [12].

As POCUS technology improves and its use increases in practice, further studies would help delineate the use of ultrasound in screening for EPN and other diseases. One comparative retrospective review of 210 EPN patients found CT to be 100% specific, while ultrasound was 69% specific and plain X-ray 65% specific [6]. A separate retrospective analysis of a prospectively collected data of 243 cases of all pyelonephritis, both uncomplicated and complicated, found abnormalities using ultrasound in 40% and 61% of cases respectively [13].

Ultrasound elastography, which provides measurement on tissue stiffness, may be of use in the future to aid in diagnosing pathology of native kidney disease [14]. Elastography research and clinical applications are increasing and we may be seeing its use more in the POCUS arena soon as well. Additionally, contrast enhanced ultrasound may detect small abnormalities in renal parenchyma with comparable sensitivity and specificity to CT scan without the risk of radiation exposure [14]. This may be especially useful in patients with poor renal function such as our patient who are at risk for further nephropathy.

Limitations in POCUS include nearby bowel gas, which can cause confounding shadowing or obscuring the view. POCUS does not allow for categorizing the type and extent of emphysematous pyelonephritis as seen on CT scan. Additionally, POCUS is user dependent and varies based on training experience. POCUS is useful in resource-limited settings worldwide because of its portability and size [15]. Because of the rapid growth of POCUS internationally, an international curriculum was published by the International Federation for Emergency Medicine Point of Care Ultrasound in 2015 to meet the critical and growing need for a standardized approach to international POCUS use [16]. Guidelines for the use of POCUS in emergency medicine have been published for almost 2 decades in the United States of America with the most recent update occurring in 2016 [17, 18].

## 4. Conclusion

POCUS has become an exciting and useful adjunct in the rapid evaluation of critical patients due to its accessibility, rapid access, and cost effectiveness. Diagnoses such as emphysematous pyelonephritis have characteristic findings and physician's using POCUS can recognize the characteristic signs of gas in the parenchyma of the kidney and/or bladder, alerting the physician to the critical diagnosis of EPN. Quick recognition of abnormal ultrasound findings representing disease processes in the appropriate setting can lead to the correct diagnosis and/or decrease time to diagnosis resulting in more rapid and correct intervention.
